# Neutrophil-to-Lymphocyte Ratio as an Independent Predictor of In-Hospital Mortality in Patients with Acute Intracerebral Hemorrhage

**DOI:** 10.3390/medicina57060622

**Published:** 2021-06-15

**Authors:** Răzvan Alexandru Radu, Elena Oana Terecoasă, Cristina Tiu, Cristina Ghiță, Alina Ioana Nicula, Andreea Nicoleta Marinescu, Bogdan Ovidiu Popescu

**Affiliations:** 1 “Carol Davila” University of Medicine and Pharmacy Bucharest, 020021 Bucharest, Romania; raduarazvan@yahoo.com (R.A.R.); cristinatiu@yahoo.com (C.T.); alinapavel74@yahoo.com (A.I.N.); andreea_marinescu2003@yahoo.com (A.N.M.); bogdan_ovidiu_popescu@yahoo.com (B.O.P.); 2 Department of Neurology, University Emergency Hospital Bucharest, 050098 Bucharest, Romania; cristina.ghita8@yahoo.com; 3 Department of Radiology and Medical Imaging, University Emergency Hospital Bucharest, 050098 Bucharest, Romania; 4 Department of Neurology, Colentina Clinical Hospital, 020125 Bucharest, Romania; 5 Laboratory of Cell Biology, Neurosciences and Experimental Myology, ‘Victor Babes’ National Institute of Pathology, 050096 Bucharest, Romania

**Keywords:** intracerebral hemorrhage, neutrophil-to-lymphocyte ratio, in-hospital mortality, outcome

## Abstract

*Background and Objectives*: Neutrophil-to-lymphocyte ratio (NLR), a very low cost, widely available marker of systemic inflammation, has been proposed as a potential predictor of short-term outcome in patients with intracerebral hemorrhage (ICH). *Methods*: Patients with ICH admitted to the Neurology Department during a two-year period were screened for inclusion. Based on eligibility criteria, 201 patients were included in the present analysis. Clinical, imaging, and laboratory characteristics were collected in a prespecified manner. Logistic regression models and receiver operating characteristics (ROC) curves were used to assess the performance of NLR assessed at admission (admission NLR) and 72 h later (three-day NLR) in predicting in-hospital death. *Results:* The median age of the study population was 70 years (IQR: 61–79), median admission NIHSS was 16 (IQR: 6–24), and median hematoma volume was 13.7 mL (IQR: 4.6–35.2 mL). Ninety patients (44.8%) died during hospitalization, and for 35 patients (17.4%) death occurred during the first three days. Several common predictors were significantly associated with in-hospital mortality in univariate analysis, including NLR assessed at admission (OR: 1.11; 95% CI: 1.04–1.18; *p* = 0.002). However, in multivariate analysis admission, NLR was not an independent predictor of in-hospital mortality (OR: 1.04; 95% CI: 0.9–1.1; *p* = 0.3). The subgroup analysis of 112 patients who survived the first 72 h of hospitalization showed that three-day NLR (OR: 1.2; 95% CI: 1.09–1.4; *p* < 0.001) and age (OR: 1.05; 95% CI: 1.02–1.08; *p* = 0.02) were the only independent predictors of in-hospital mortality. ROC curve analysis yielded an optimal cut-off value of three-day NLR for the prediction of in-hospital mortality of ≥6.3 (AUC = 0.819; 95% CI: 0.735–0.885; *p* < 0.0001) and Kaplan–Meier analysis proved that ICH patients with three-day NLR ≥6.3 had significantly higher odds of in-hospital death (HR: 7.37; 95% CI: 3.62–15; log-rank test; *p* < 0.0001). *Conclusion*: NLR assessed 72 h after admission is an independent predictor of in-hospital mortality in ICH patients and could be widely used in clinical practice to identify the patients at high risk of in-hospital death. Further studies to confirm this finding are needed.

## 1. Introduction

Intracerebral hemorrhage (ICH) is the second leading cause of stroke worldwide and accounts for roughly one-fourth of all strokes. It is estimated that ICH causes nearly 50% of all stroke-related deaths [1,2]. Many new technological advances have shown promising results in exploratory trials for identifying therapies capable of modifying outcomes of patients with ICH. However, none of these have been proven effective in phase 3 trials, and the ideal patient to be selected for different novel surgery techniques is not yet identified. The same uncertainties also apply to the role of secondary prevention strategies directed against the different pathophysiological pathways involved in hematoma and edema expansion [3].

All components of the ICH score (ICH volume, Glasgow Coma Scale, age over 80, infratentorial origin of ICH, and presence of intraventricular blood) are well-known independent prognostic factors of short-term mortality [4]. Recently, hematoma growth has also been identified as a major, potentially modifiable, driver of outcome, and a few factors associated with early hematoma expansion have been identified [5]. Searching for better predictors of short-term mortality is imperatively needed as ICH is still characterized by a very severe prognosis. Most deaths accounting for the high short-term mortality occur during the first week of hospitalization, and therefore better means of identifying the ICH patients prone to have an unfavorable short-term prognosis will be helpful to guide the selection algorithm of trials, addressing the benefits of different neurosurgical or aggressive medical strategies in ICH [6]. Elucidating the individual role of several potentially modifiable biological factors predicting the short-term ICH mortality may enhance survival given the constantly improving standards of care and the expected potential of new therapies [7].

Neuroinflammation produced by the initial brain insult in ICH impacts outcome, and inflammatory markers are thought to have a crucial role in secondary brain injury in ICH patients [8]. Neutrophil-to-lymphocyte ratio (NLR) is an essential marker of inflammatory response and a readily available measure in hospitalized patients. NLR was previously shown by some studies to be associated with ICH volume, hematoma expansion, and short-term mortality [9,10], and different meta-analyses have tried to establish the strength of association between admission NLR and functional outcome in ICH patients. However, they yielded conflicting results, and the matter is still under debate [11]. Theoretically, NLR could be a helpful link between systemic and intracerebral inflammation and could identify patients at risk of bad outcomes. As opposed to other severe conditions, NLR assessed close to the moment of ICH onset shows relatively low median values, and a recent study has indicated that subsequent NLR determination, during the first week of hospitalization, may be a much better predictor of outcome [12]. Using NLR as a marker of disease severity in patients with ICH is attractive as it is a widely available and very low cost biomarker. The present study aims to determine the utility of admission NLR and three-day NLR in predicting in-hospital death in patients with ICH.

## 2. Materials and Methods

All patients with intracerebral hemorrhage admitted to the Neurology Department of the University Emergency Hospital Bucharest between July 2018 and July 2020 were screened for inclusion in this retrospective observational cohort study. We defined the following exclusion criteria for our initial cohort: (1) lack of signed informed consent forms; (2) admission likelihood of survival beyond three days considered very low; (3) initial admission in the ICU; (4) subsequent surgery or shunt placement; (5) inter-hospital transfers. For this study, the following additional exclusion criteria were used: (6) hemorrhage from a colocalized tumor; (7) traumatic ICH; (8) ischemic stroke with hemorrhagic transformation or aneurysmal SAH with parenchymal hematoma; (9) associated hematologic disorder; (10) associated malignancy; (11) missing follow-up data; (12) presence of an apparent infection on arrival; (13) steroid treatment; (14) time interval between ICH onset and first CT scan >24 h; (15) complete blood count (CBC) at admission not available. If the exact time of stroke onset was not available, the time when the subject was last seen normal was substituted only if it was <24 h. 

Demographic and clinical characteristics were collected from the medical records and electronic health system. The attending neurologist determined Glasgow Coma Scale (GCS) and NIHSS (National Institute of Health Stroke Scale) scores on admission. In-hospital death from any cause was used as an outcome measure. Traditional risk factors definitions were detailed in a previously published protocol for our cohort [13]. Admission complete blood count (CBC) was conducted on an automated hematology analyzer (MS9/5s, Melet Schloesing Laboratoires, Osny, France). Neutrophil-to-lymphocyte ratio at admission (admission NLR) and 72 h later (3-day NLR) was calculated as the absolute neutrophil counts divided by the absolute lymphocyte counts. 

CT scans performed at admission and during hospitalization were exported from the hospital picture archiving and communication system and imported in Analyze 14 (AnalyzeDirect, Overland Parc, KS, USA). CT scans were analyzed blinded to date and clinical outcomes. All hematoma measurements were made by R.A.R. Hematoma volume (HV) was measured by manually segmenting each slice with an automatic trace function with threshold values between 44 and 80 HU. Artifacts were excluded if needed with the help of the draw limit function. Further image analysis was performed with Horos (Horosproject.org) by R.A.R. two weeks after finishing the initial volume measurements. Hematoma location was defined using The Cerebral Hemorrhage Anatomical Rating Instrument (CHARTS). For this study, lobar and deep hemorrhages were considered together with the uncertain origin category [14]. HVs were also assessed with the ABC/2 method as these values were needed for computing the ICH score [4,15]. Presence of IVH was noted, and IVH severity was determined with the GRAEB score [16].

All statistical analyses were performed using NCSS 12 Statistical Software (NCSS, LLC. Kaysville, UT, USA) and MedCalc 18.11.3 Statistical Software (MedCalc Software, Ostend, Belgium). Continuous variables are described as median and 25–75 IQR, and categorical variables are presented as absolute numbers and percentages. For comparisons between continuous variables, we used Mann–Whitney or Kruskal–Wallis tests, according to the number of selected groups analyzed. For testing the strength of association between categorical variables, we used the chi-squared test or Fisher’s exact test, depending on the number of analyzed cases. Multivariate analysis was performed using logistic regression and taking into account full models that included variables achieving a *p*-value ≤ 0.1 in univariate analysis. Subsequently, a predictive modelling strategy with the backward stepwise method of entering data was used. The goodness-of-fit of the logistic regression models was assessed with the Hosmer–Lemeshow test. Receiver operating characteristics (ROC) curve analysis was used to analyze the performance of 3-day NLR in predicting in-hospital death, and the Youden index to establish the optimal cut-off value for prediction of in-hospital death. Survival analysis was performed using Kaplan–Meier method, and the log-rank test was used to statistically compare the curves between the predefined groups of patients. A preset significance level of *p* < 0.05 was considered for all comparisons.

## 3. Results

### 3.1. NLR Assessed at Admission

A total of 383 patients were admitted to the Department of Neurology with ICH diagnosis during the study period. One hundred and fifty-six patients were excluded from the study based on established eligibility criteria: 43 patients were admitted more than 24 h after symptoms onset, 7 patients were transferred from other hospitals, 21 patients did not have signed informed-consent forms, 18 patients underwent a subsequent neurosurgery intervention, 26 were directly admitted to the ICU, 21 patients had incomplete admission blood analyses in the hospital records, 24 patients had missing follow-up data, and 22 patients were excluded due to other exclusion criteria. As a result, the final study population included 201 patients. 

Table 1 shows the baseline and clinical characteristics of the study population. The median age of the included patients was 70 years, and 111 patients, representing 55.2% of our study population, were male. Median admission HV was 13.7 mL, and median ICH score was 1 point. Out of the 201 patients, 90 patients (44.8%) died during hospitalization. As compared to the group of patients discharged alive, those who died were older (73 years vs. 66 years; *p* = 0.001) and more frequently diagnosed with diabetes mellitus (47.8% vs. 24.3%; *p* < 0.001) or coronary artery disease (14.4% vs. 3.6%; *p* = 0.009). All clinical severity scores showed a strong association with ICH mortality: median ICH score (2 vs. 1; *p* < 0.001), median NIHSS score (25 vs. 10; *p* < 0.001), and median GCS score (9.5 vs. 15; *p* < 0.001). ICH patients who died during hospitalization were also more likely to have higher admission HV (27.8 mL vs. 7.3 mL; *p* < 0.001), and ventricular effraction was more frequently seen in this group (62.1% vs. 31.2%; *p* < 0.001). Admission blood glucose levels were significantly higher for patients who died during hospitalization (176 mg/dL vs. 126 mg/dL; *p* < 0.001). In univariate analysis, the following complete blood count parameters were associated with in-hospital death: leukocytes (12130 cells/mm^3^ vs. 9400 cells/mm^3^; *p* < 0.001), neutrophils (9145 cells/mm^3^ vs. 6600 cells/mm^3^; *p* < 0.001), admission NLR (5.6 vs. 3.7; *p* = 0.002), and three-day NLR (9.7 vs. 4.6; *p* < 0.001). 

Variables achieving a *p*-value of ≤0.1 in univariate analysis were used to fit a full model of logistic regression analysis, with the following exceptions: (1) NIHSS score was included as sole clinical severity score and NLR as a pooled measure of complete blood count markers, to reduce redundancy; (2) dyslipidemia and hemoglobin (Hb) values were not included due to the questionable clinical significance of the results; (3) ICH score was not included due to potential collinearity with its component parameters. As a result, the final model included the following variables: age, admission NIHSS score, coronary artery disease, ICH volume at admission, GRAEB score, GFR rate, admission blood glucose, NLR at admission. *p*-value and chi-squared in Hosmer–Lemeshow goodness-of-fit test were 0.9 and 3.1, suggesting that the model fitted reasonably well. The results are summarized in Table 2.

Factors increasing the odds of in-hospital death were age (OR: 1.07; 95% CI: 1.03–1.11), admission NIHSS score (OR: 1.13; 95% CI: 1.07–1.19), admission ICH volume (OR: 1.02; 95% CI: 1.01–1.05), and admission blood glucose levels ≥ 180 mg/dL (OR: 3.9; 95% CI: 1.4–10.5). NLR at admission lost statistical significance following multivariate analysis (OR: 1.04; 95% CI: 0.9–1.1; *p* = 0.3). Subsequently, a predictive modeling strategy was used (backward stepwise regression), which confirmed the performance of a model including age, admission NIHSS score, admission HV, and blood glucose levels ≥ 180 mg/dL in predicting in-hospital death.

### 3.2. NLR Assessed 72 h after Admission

To establish the value of NLR assessed 72 h after admission (three-day NLR) for predicting in-hospital death, we reanalyzed the initial study population and selected the patients who survived the first 72 h of hospitalization and had a CBC analysis determined 72 h ± 6 h after the initial CBC. As 35 patients (17.4%) died during the first three days of hospitalization and 54 patients (26.8%) did not have a CBC at this time point, the study population included in this analysis consisted of 112 patients. Of these patients, 76 (67.9%) were alive at three days. In univariate analysis, clinical and imaging factors that remained significant predictors of in-hospital mortality for these patients were age (median 75 vs. 66.5; *p* = 0.01), presence of diabetes mellitus (47.2% vs. 23.7%; *p* = 0.01), coronary artery disease (13.9% vs. 1.32%, *p* = 0.005), admission ICH score (median 2 vs. 1, *p* < 0.001), admission NIHSS score (median 20 vs. 12; *p* < 0.001), admission GCS (median 12 vs. 15; *p* < 0.001), admission HV (27.3 mL vs. 11.5 mL; *p* <0.001), presence of ventricular effraction (61.1% vs. 34.2%; *p* = 0.007), admission GRAEB score (median 1.5 vs. 1; *p* = 0.004), admission blood glucose >180 mg/dL (176.5 mg/dL vs. 137 mg/dL; *p* < 0.001), leukocytes count (12,545/mm^3^ vs. 9860/mm^3^; *p* < 0.001), neutrophils count (9750/mm^3^ vs. 6925/mm^3^; *p* < 0.001), admission NLR (6.5 vs. 4.4; *p* = 0.002), and three-day NLR (9.9 vs. 4.6; *p* < 0.001). Detailed characteristics of this study population are presented in Supplementary Table S1.

As in-hospital death was observed for 36 patients in the population of patients surviving the first 72 h of hospitalization, we performed multivariate statistics using a full model that only included the following independent variables: age, admission NIHSS score, admission HV, and three-day NLR. Age (OR: 1.05; 95% CI: 1.007–1.1; *p* < 0.02) and three-day NLR (OR: 1.2; 95% CI: 1.09–1.4; *p* < 0.001) remained independent predictors for in-hospital death in this population. The results are summarized in Table 3. 

**Figure 1:**
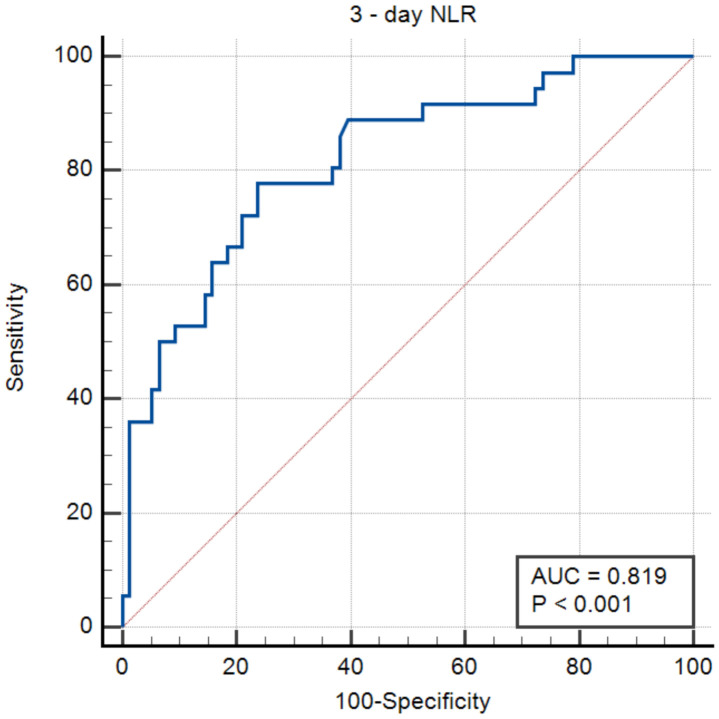
ROC curve of three-day NLR to predict in-hospital death for ICH patients surviving first 72 h of hospitalization. Youden index = 0.54 with associated criterion three-day NLR ≥ 6.3. AUC = 0.819; 95% CI: 0.735–0.885; *p* < 0.0001. Sensitivity: 77.8%. Specificity: 76%. Positive predictive value: 59.6%; negative predictive value: 87.7%.

**Figure 2:**
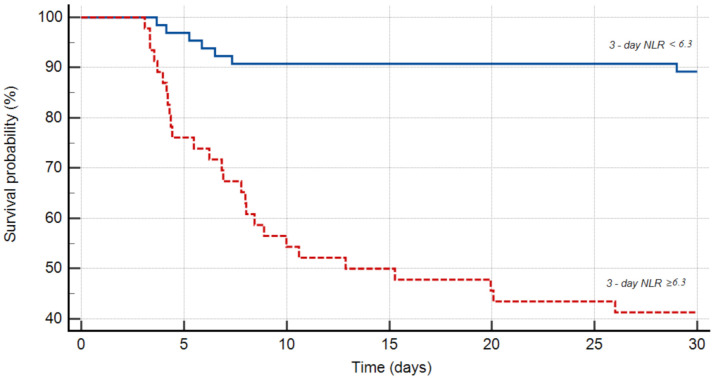
Kaplan–Meier curve showing 30-day mortality in subjects surviving first 72 h of hospitalization, according to NLR < 6.3 (solid blue line; *n* = 65 patients) vs. NLR ≥ 6.3 (dotted red line; *n* = 47 patients).

In the receiver operating characteristic curve analysis, the optimal cut-off value of three-day NLR for prediction of in-hospital death among patients surviving the first 72 h of hospitalization was found to be 6.3 with a sensitivity of 77.8% and a specificity of 76.3% (area under the curve: 0.819; 95% CI: 0.735–0.885; *p* < 0.0001; Figure 1). The Kaplan–Meier analysis showed that patients surviving the first 72 h of hospitalization with three-day NLR ≥ 6.3 had significantly higher 30-day mortality than those with three-day NLR < 6.3 (HR: 7.37; 95% CI: 3.62–15; log-rank test; *p* <0.0001; Figure 2).

## 4. Discussion

This study aimed to analyze the relationship between NLR assessed at admission and 72 h later and in-hospital mortality in patients with ICH. Several prior reports yielded conflicting results about the usefulness of NLR as an independent predictor of in-hospital death in ICH, and therefore the role of admission NLR as an outcome predictor in ICH patients is still under debate [10]. Our study results show that even if the admission NLR values are higher in ICH patients who die during hospitalization, this parameter loses its significance after adjustment for well-known factors associated with higher odds of in-hospital ICH mortality and therefore cannot be considered an independent predictor of in-hospital death in ICH patients. In our cohort, age, NIHSS score at admission, blood glucose levels >180 mg/dL, and hematoma volume were independent predictors of outcome. The most recent (and one of the few available) meta-analysis on this topic supports our findings by showing that admission NLR is not an independent predictor of in-hospital mortality in ICH; however, due to the scanty studies addressing this question, the meta-analysis included only two studies and the heterogeneity was high [11].

NLR is a reflection of physiological stress and a marker of the interaction between different immune pathways. NLR was shown to be an independent predictor of in-hospital mortality for many cardiovascular, neurovascular, and intensive-care conditions [17,18], and high NLR values can be observed in very heterogenous diseases and conditions. This is why NLR is generally considered a marker of physiologic stress among hospitalized patients. NLR is a widely available, very low cost investigation and therefore, if its usefulness for assessing short-term ICH prognosis could be clearly demonstrated, its importance for daily practice could be very high.

The findings of our study indicate that the time interval between ICH onset and moment of NLR assessment is important in determining the relationship between this ratio and short-term outcome in patients with ICH. In line with our results, which showed that only the ratio assessed 72 h after admission and not the one assessed at the time of admission is an independent predictor of in-hospital mortality, others have found a better correlation between NLR calculated 24 h after admission or at different time moments during the first week of hospitalization and short-term ICH mortality [9,12]. These findings could have several explanations. First, if this ratio is calculated 24 h after admission or even later, patients with very high hematoma volumes or very high ICH scores, indicative of a high probability of early death, are automatically excluded. Second, initial peripheral immune activation is observed approximately six hours after experimentally induced ICH. In our study, the median time interval between stroke onset and first assessment of NLR (admission NLR) was 4.3 h (25–75 IQR: 2.2–11.1 h), and other authors have reported median ICH onset to NLR assessment time intervals of ≈6 h [9,19]. Thus, calculation of this ratio at the moment of hospital admission might be too early to identify any peripheral immune changes induced by the ICH and might reflect the effects of the neural–immune crosstalk only on its initial upslope, thereby limiting its value as a predictor of in-hospital mortality and short-term outcome.

The immune and nervous systems maintain a homoeostasis under normal physiological conditions. An acute ICH interrupts this balance and leads to initial activation of peripheral inflammatory cells and secondary brain inflammation. This response, initially adaptive, soon becomes maladaptive, potentially contributing to the expansion of ICH-associated lesions and the worsening of the neurologic condition, creating the premise for a secondary immune suppression phase induced by the brain, which is likely associated with a rise in post-stroke infections. Ref. [20] Thus, it can be postulated that dynamic NLR values might reflect this crosstalk. In our study, most of the patients who survived the first 72 h of hospitalization and had a three-day NLR ≥6.3 died during the first two weeks (Figure 2), potentially indicating that a severe initial peripheral inflammatory response is associated with further brain injury. Furthermore, lower three-day NLR was associated with better outcomes, perhaps reflecting an already better control of inflammatory pathways. Further studies should address the impact of inflammatory biomarker dynamics in this brain–immune crosstalk.

One in three ICH patients will die during the first 30 days, but a gradual decline in the overall ICH fatality rate has been reported during the last 27 years. However, the proportion of patients admitted in a coma and the very early <48 h case fatality rate has remained unchanged during this time period (≈43%). Ref. [21] This factor likely explains the heterogeneity of different studies focusing on factors like NLR to identify patient subgroups that would likely benefit from additional therapeutic options. The lack of success in modifying outcomes in patients with very large hematomas makes any potential predictor, apart from those included in the commonly used prognostic scores, unlikely to yield any consistent results [3]. The initial report of the ICH score published in 2001 ascribed mortality rates of 97% and 72% for ICH scores of 5 and 4 points, respectively, and these percentages seem to still be valid nowadays, in more performant ICU settings [4,22]. This is why future analysis of predictors of outcome in patients with ICH should probably focus on populations with less severe ICH scores at admission and less dramatic clinical course.

Several findings of our study warrant a short discussion. First, in univariate analysis, dyslipidemia was significantly more frequent among our ICH patients discharged alive. In many previous studies and meta-analyses, low serum low-density cholesterol (LDLc) levels were shown to be associated with an increased risk of ICH and a higher risk of hematoma expansion and mortality [23,24], which might be in line with our findings. However, we used a composite measure of previous statin therapy, low LDLc values, and high triglyceride values to define dyslipidemia in our study, and for some patients who died during the first hours of hospitalization, LDLc values were not available, so this result must be judged with caution. Second, in our cohort, Hb levels were significantly lower in patients who died during hospitalization. Similar findings were previously reported, and a recent meta-analysis observed a dose–response relationship between Hb levels up to 14 mg/dL and decreased survival in ICH patients. Beyond this cut-off limit, the strengths of the relationship was lost [25]. Given the low sample size of our study and our median Hb level of 13.8 mg/dL, we decided to not include Hb levels in multivariate analysis. Further analysis of these findings is necessary. 

The current study has several limitations. First, the small sample size and its retrospective design prohibit the delineation of any potentially causal relationships. Second, some patients admitted with the diagnosis of ICH in our hospital were not included in our analyses, as the study was restricted to patients hospitalized in the Stroke Unit or Neurology Ward, missing those directly admitted in the ICU and those who underwent emergency neurosurgical procedures, including extraventricular drain placement. Third, CBC analysis determined 72 ± 6 h after the initial CBC was not available for 54 patients who survived the first 72 h of hospitalization, and therefore these patients were not included in the specific subgroup analysis, leading to a potential selection bias. However, the results of our study and their similarity with other reported findings on the same topic suggest that NLR assessed during the first few hours after ICH onset cannot be used as a predictor of in-hospital mortality while NLR assessed 72 h later is an independent predictor of in-hospital death in ICH patients and might be useful in clinical practice. The Kaplan–Meier curves showed an overall low survival in patients with three-day NLR values ≥6.3 mg/dL, and this cut-off point is in line with previously reported NLR values associated with a low probability of 30-day survival and higher odds of poor outcome [26].

## 5. Conclusions

NLR assessed 72 h after admission is an independent predictor of in-hospital mortality in ICH patients and could be widely used in clinical practice as an additional biomarker aimed to identify the patients at high risk of in-hospital death. Further studies are required to determine whether specific interventions in patients with high NLR values at different time points after ICH onset can modify the short-term outcomes of this devastating disease.

## Figures and Tables

**Table 1:** Demographic, clinical, and imaging characteristics of the study population.

Parameter (Unit)	Overall *n* = 201 (100%)	Alive at Discharge *n* = 111 (55.2%)	In-Hospital Death *n* = 90 (44.8%)	*p*
Age (median, 25–75 IQR)	70 (61–79)	66 (56–75)	73 (63–81.25)	0.001
Gender (*n*, % male)	111 (55.2%)	60 (54.1%)	51 (45.9%)	0.7
Smokers	33 (16.4%)	17 (15.3%)	17 (18.9%)	0.5
Chronic alcohol intake	48 (23.8%)	28 (25.2%)	20 (22.2%)	0.6
Medical History (*n*, %)				
Previous Ischemic Stroke	7 (3.5%)	4 (3.6%)	3 (3.3%)	0.9
Previous Hemorrhagic Stroke	27 (13.4%)	18 (16.2%)	9 (10%)	0.2
Previous Known Hypertension	154 (76.5%)	89 (80%)	65 (72%)	0.2
Diabetes mellitus	50 (25%)	27 (24.3%)	43 (47.8%)	<0.001
Dyslipidemia	122 (60.7%)	82 (73.8%)	40 (44.4%)	<0.001
Atrial fibrillation	27 (13.47%)	13 (11.8%)	14 (15.6%)	0.4
Coronary artery disease	17 (8.5%)	4 (3.6%)	13 (14.4%)	0.009
Previous treatment (*n*, %)				
Previous antiplatelet treatment	52 (25.8%)	29 (26.1%)	23 (25.6%)	0.9
Previous anticoagulant treatment	25 (12.44%)	10 (9.01%)	15 (16.7%)	0.1
Previous antihypertensive treatment	186 (51%)	54 (48.6%)	49 (54.4%)	0.3
Clinical ICH severity (median, 25–75 IQR)				
ICH score	1 (0–2)	1 (0–1)	2 (1–3)	<0.001
Admission NIHSS	16 (6–24)	10 (4–17)	25 (16–36)	<0.001
Admission GCS	15 (10–15)	15 (15–15)	9.5 (3–14)	<0.001
Pre-Stroke mRS	0 (0–1)	0 (0–1)	0 (0–1)	0.4
ICH imaging				
Hematoma Volume (mL, 25–75 IQR)	13.7 (4.6–35.2)	7.3 (1.8–19.9)	27.8 (11.2–63.7)	<0.001
HV < 10 mL	80 (41%)	62 (57.4%)	18 (20.7%)	<0.001
HV 10–29 mL	58 (29.7%)	30 (27.8%)	28 (32.2%)	
HV 30–49 mL	27 (13.8%)	11 (10.2%)	16 (18.4%)	
HV ≥ 50 mL	30 (15.3%)	5 (4.6%)	25 (28.7%)	
Lobar hemorrhage (*n*, %)	50 (24.8%)	25 (22.5%)	25 (27.8%)	0.4
Deep hemorrhage (*n*, %)	140 (69.6%)	81 (72.9%)	59 (65.5%)	0.2
Ventricular effraction (*n*, %)	90 (44.9%)	35 (31.2%)	56 (62.1%)	<0.001
GRAEB score (median, 25–75 IQR)	0 (0–4)	0 (0–1)	2 (0–8)	<0.001
Admission Blood Analyses (median, 25–75 IQR)				
Creatinine Clearance (mL/min/1.73 m^2^)	79 (55.3–91.4)	80.8 (59.8–93.1)	73.5 (49.4–89.5)	0.07
Glucose (mg/dL)	141 (110.5–191.5)	126 (104–153)	176.5 (132–217)	<0.001
Glucose > 180 mg/dL (*n*, %)	56 (27.8%)	16 (14.4%)	40 (44.4%)	<0.001
WBC (cells/mm^3^)	10,040 (7900–12,950)	9400 (7820–11,400)	12,130 (8310–14,922)	<0.001
NEUT (cells/mm^3^)	7200 (5050–10,250)	6600 (4900–8600)	9145 (5750–12,500)	<0.001
LYMPHs (cells/mm^3^)	1600 (1105–2300)	1700 (1200–2400)	1550 (1000–2300)	0.4
MONOs (cells/mm^3^)	600 (400–885)	600 (400–800)	605 (475–900)	0.1
HGB (g/dL)	13.8 (12.5–14.9)	13.9 (12.6–15.4)	13.4 (12.2–14.7)	0.05
PLTs (cells × 1000/mm^3^)	209 (162–268)	213 (167–267)	208 (160–270)	0.9
INR	1.1 (1.04–1.2)	1.1 (1.04–1.18)	1.1 (1.06–1.39)	0.01
Admission NLR	4.3 (2.6–7.8)	3.7 (2.4–6)	5.6 (2.9–9.8)	0.002

**Table 2:** Factors associated with in-hospital mortality in ICH patients (multivariate statistics).

	Univariate Analysis		Multivariate Analysis	
	*p-*Value	OR (95% CI)	*p-*Value	OR (95% CI)
Age	0.001	1.03 (1.01–1.06)	<0.001	1.07 (1.03–1.11)
Admission NIHSS	<0.001	1.15 (1.1–1.2)	<0.001	1.13 (1.07–1.19)
Coronary artery disease	0.009	4.5 (1.4–14.3)	0.06	4.6 (0.9–23.5)
Admission HV	<0.001	1.03 (1.02–1.05)	0.02	1.02 (1.01–1.05)
Admission GRAEB score	<0.001	1.2 (1.1–1.4)	0.1	1.1 (0.9–1.2)
Creatinine Clearance	0.07	0.99 (0.97–1)	0.7	1.01 (0.9–1.01)
Glucose >180 mg/dL	<0.001	4.7 (2.3–8.7)	0.006	3.9 (1.4–10.5)
Admission NLR	0.002	1.1 (1.04–1.18)	0.3	1.04 (0.9–1.1)

**Table 3:** Factors associated with in-hospital mortality in ICH patients surviving first 3 days of hospitalization (multivariate statistics).

	Univariate Analysis		Multivariate Analysis	
	*p-*Value	OR (95% CI)	*p-*Value	OR (95% CI)
Age	0.01	1.05 (1.02–1.08)	0.02	1.05 (1.007–1.1)
Admission NIHSS	<0.001	1.12 (1.07–1.2)	0.06	1.06 (0.9–1.1)
Admission HV	<0.001	1.03 (1.01–1.04)	0.07	1.02 (0.9–1.04)
Three-day NLR	<0.001	1.3 (1.1–1.4)	<0.001	1.2 (1.09–1.4)

## Data Availability

Data used in this study may be provided by the corresponding author upon reasonable requests.

## Supplementary Materials

The following are available online at https://www.mdpi.com/article/10.3390/medicina57060622/s1: Table S1: Demographic, clinical, and imaging characteristics of patients surviving at three days.
