# Supplementary material for: Neutrophil-to-Lymphocyte Ratio as an Independent Predictor of In-Hospital Mortality in Patients with Acute Intracerebral Hemorrhage
Source: Medicina (Kaunas). 2021 Jun 15;57(6):622. doi: 10.3390/medicina57060622 (PMC8232097; doi:10.3390/medicina57060622)
Supplement: Supplementary file 1 [file medicina-57-00622-s001.zip › medicina-1248066-supplementary.pdf]

Supplementary Table S1. Demographic, Clinical and Imaging Characteristics of patients surviving at three days			
**Parameter (Unit)**	**Alive at Discharge (n = 76, 67.9%)**	**In-Hospital Death (n = 36, 32.1%)**	**p**
Age (years, median)	66.5	75	0.01
Gender (n,% male)	45 (59.21%)	23 (63.89%)	0.6
Smokers (n, %)	14 (18.42%)	9 (25%)	0.5
Chronic alcohol intake (n, %)	23 (30.26%)	10 (27.78%)	0.7
Medical History (n, column%)			
Previous Ischemic Stroke	3 (3.9%)	2 (5.5%)	0.7
Previous Hemorrhagic Stroke	11 (14.47%)	5 (13.89%)	0.9
Previous Known Hypertension	59 (78.67%)	27 (77.14%)	0.85
Diabetes mellitus	18 (23.68%)	17 (47.22%)	0.01
Dyslipidemia	59 (77.63%)	20 (55.56%)	0.01
Atrial fibrillation	8 (10.6%)	5 (15.1%)	0.5
Coronary artery disease	1 (1.32%)	5 (13.89%)	0.005
Previous treatment (n, %)			
Previous antiplatelet treatment	21 (27.63%)	8 (22.22%)	0.5
Previous anticoagulant treatment	7 (9.21%)	4 (11.11%)	0.7
Previous antihypertensive treatment	34 (47.22%)	17 (53.13%)	0.5
Clinical ICH severity (median, 25-75 IQR)			
ICH score	1 (0-1)	2 (1-3)	<0.001
Admission NIHSS	12 (4-18)	20 (10-26)	<0.001
Admission GCS	15 (14-15)	12 (8-15)	<0.001
Pre-Stroke mRS	0 (0-0)	0 (0-1)	0.4
ICH imaging			
Admission HV (ml, 25-75 IQR)	11.5 (2.3-24.8)	27.3 (11.8-59.6)	<0.001
Lobar hemorrhage (n,%)	18 (23.68%)	12 (33.33%)	0.2
Deep hemorrhage(n,%)	56 (73.68%)	23 (63.89%)	0.2
Ventricular effraction (n, %)	26 (34.21%)	22 (61.11%)	0.007
Admission GRAEB score (median, 25-75 IQR)	0 (0-1)	1.5 (0-6)	0.004
Admission Blood-Analyses			
Creatinine Clearance (ml/min/1.73m^2)	82.3 (59.1-94.9)	73.2 (52.7-93.3)	0.3
Glucose mg/dl (median, 25-75 IQR)	137 (105-162)	176.5 (137-207)	<0.001
Glucose > 180 mg/dl (n,%)	12 (15.79%)	16 (44.44%)	0.001
WBC (cells/mm^3)	9860 (7547-11845)	12545 (9865-15195)	0.001
NEUT (cells/mm^3)	6925 (4900-9475)	9750 (6825-12500)	<0.001
LYMPHs (cells/mm^3)	1500 (1100-2200)	1500 (1200-2275)	0.9
MONOs (cells/mm^3)	600 (400-800)	664 (500-900)	0.2
HGB g/dl (median, 25-75 IQR)	13.8 (12.6 – 15.3)	13.3 (12.4 – 14.7)	0.3
PLTs (cells/mm^2)	197000 (156500-250750)	206000 (150000-272000)	0.7
INR (median, 25-75 IQR)	1.1 (1.04 – 1.2)	1.1 (1.06 – 1.3)	0.1
Admission NLR (median, 25-75 IQR)	4.4 (2.4-7.6)	6.5 (3.6-11.3)	0.002
3-day NLR (median, 25-75 IQR)	4.6 (3.3-6.2) 76p	9.9 (6.5-15.5)	<0.001
